# Quinoline Antimalarials Increase the Antibacterial Activity of Ampicillin

**DOI:** 10.3389/fmicb.2021.556550

**Published:** 2021-06-02

**Authors:** Olajumoke A. Olateju, Chinedum P. Babalola, Olujide O. Olubiyi, Olayinka A. Kotila, David A. Kwasi, Anderson O. Oaikhena, Iruka N. Okeke

**Affiliations:** ^1^Department of Pharmaceutical Chemistry, Faculty of Pharmacy, University of Ibadan, Ibadan, Nigeria; ^2^Centre for Drug Discovery Development and Production (CDDDP), Faculty of Pharmacy, University of Ibadan, Ibadan, Nigeria; ^3^Department of Pharmaceutical Chemistry, Faculty of Pharmacy, Obafemi Awolowo University, Ile-Ife, Nigeria; ^4^Department of Pharmaceutical Microbiology, Faculty of Pharmacy, University of Ibadan, Ibadan, Nigeria

**Keywords:** penicillins, chloroquine, quinine, ampicillin, drug combination, paper strip diffusion, modified disc diffusion checkerboard

## Abstract

Bacterial and malaria co-infections are common in malaria endemic countries and thus necessitate co-administration of antibiotics and antimalarials. There have long been anecdotal clinical reports of interactions between penicillins and antimalarial agents, but the nature and mechanisms of these interactions remain to be investigated. In this study, we employed antimicrobial interaction testing methods to study the effect of two antimalarials on the antibacterial activity of ampicillin *in vitro.* Paper strip diffusion, a modified disc diffusion and checkerboard methods were used to determine the nature of interactions between ampicillin and quinoline antimalarials, chloroquine and quinine, against Gram-positive and Gram-negative bacteria. The impact of antimalarials and ampicillin-antimalarial drug combinations on cell integrity of test bacteria were determined by measuring potassium release. The tested antimalarials did not show substantial antibacterial activity but quinine was bactericidal at high concentrations. Chloroquine and quinine increased ampicillin activity, with increasing concentrations extending the antibacterial’s inhibition zones by 2.7-4.4 mm and from 1.1 to over 60 mm, respectively. Observed interactions were largely additive with Fractional Inhibitory Concentration Indices of >0.5-1 for all ampicillin-antimalarial combinations. Quinine and, to a lesser extent, chloroquine increase the activity of ampicillin and potentially other β-lactams, which has implications for combined clinical use.

## Introduction

The nature of interactions between antimalarials, particularly those belonging to the quinoline class, and antibiotics has been studied extensively in the past two decades (Babalola et al., 2002, 2003, 2009; Abreu et al., 2014; Falade et al., 2016). Malaria is immunosuppressive. As a result, patients with malaria often come down with other infections (Morakote and Justus, 1988; Babalola et al., 2003; Pradhan and Ghosh, 2013; Falade et al., 2016; Popoola et al., 2019). Irrespective of how common actual co-infections are, *Plasmodium* and bacterial co-infections are often presumed, resulting in very common co-administration of these two classes of drugs in sub-Saharan Africa where malaria is endemic (Falade et al., 2016; Popoola et al., 2019). For instance, a prescription survey conducted in a tertiary institution in Nigeria showed that antimalarials were the most commonly prescribed drugs and that half of the patients on antimalarials were also placed on antibiotics (Uchefunah, 2007).

Co-administration of two or more drugs is considered rational when trying to achieve a desired therapeutic objective or treat co-morbidities but the possibility of drug-drug interactions could offset these benefits by bringing about sub-therapeutic drug concentrations that could ultimately lead to treatment failure (Martinbiancho et al., 2007). For instance, penicillin antibiotics have been reported to demonstrate *in vivo* and *in vitro* interactions with certain antimalarial agents (Babalola et al., 2003, 2009; Falade et al., 2016). Documented interactions include reduction in bioavailability of penicillins (ampicillin and cloxacillin) by 40-70 % after oral co-administration with quinine and chloroquine in healthy patients (Ali, 1985; Babalola et al., 2003; Falade et al., 2016). Other studies reported similar interactions with proguanil and artesunate (Babalola et al., 2002, 2009), suggesting that pharmacokinetic drug interaction is likely occurring at the absorption phase (Palleria et al., 2013).

The quinoline antimalarials have longed been used in the treatment of malaria, especially as caused by *Plasmodium falciparum* (Ugale et al., 2017; Center for Disease Control and Prevention [CDC], 2019). Although chloroquine and quinine have been largely phased out from current malaria treatment guidelines, they are still recommended for use in some circumstances. For instance, quinine is a second-line agent in managing complicated malaria and is recommend in pregnant women in their first trimester (World Health Organization, 2015). Chloroquine is recommended by the CDC for the treatment of uncomplicated malaria, and in pregnancy, especially in the first trimester in regions without chloroquine-resistant strains such as Central America west of the Panama Canal, Haiti, the Dominican Republic, and most of the Middle East (Center for Disease Control and Prevention [CDC], 2019). Chloroquine remains an effective choice for most *P. vivax* and *P. ovale* infections. The drug has also been used off-label and in clinical trials to manage SARS-CoV-2 infections, which sometimes require administration of an antibacterial for secondary bacterial infection (Colson et al., 2020; Devaux et al., 2020; Zhonghua Jiehe He Huxi Zazhi, 2020). Furthermore, these old antimalarials have future potential as resurgence in parasites sensitive to chloroquine has been reported in some countries where partial resistance to artemisinin and partner drug resistance exist. Older antimalarials may therefore be an interim solution to antimalarial therapy prior to discovery of newer ones, for instance, cessation of chloroquine use in Malawi was followed by the re-emergence of chloroquine-susceptible malaria (Kublin et al., 2003; Frosch et al., 2011; World Health Organization, 2018).

Of more interest is the fact that chloroquine and quinine have been reported to have some antibacterial activity, albeit at high concentrations, arising from their structural similarities to (Wolf et al., 2002; Lv et al., 2007; Davidson et al., 2008; Kharal et al., 2009; Bawa et al., 2010; Achan et al., 2011; Jagadeesh et al., 2014) quinolone antibacterials.

This study attempts to clarify the nature of interactions between penicillins and quinoline antimalarials using a range of testing methods. This investigation is long overdue since the drugs in question have been in clinical use for more than half a century, often in combination. We evaluated, *in vitro*, the effect of chloroquine and quinine on the antibacterial effect of ampicillin, against ampicillin-sensitive and -resistant isolates.

## Materials and Methods

### Cultivation of Strains and Inoculum Preparation

Table 1 shows the test organisms used in the study. Isolates were maintained in Luria Broth: glycerol 1:1 at −80 °C and cultured on Muller Hinton agar (MHA; Oxoid, United Kingdom) at 37°C overnight prior to use. To prepare bacterial suspensions, three morphologically similar colonies from each respective agar plate were suspended in 4 mL of 0.9 %w/v saline (BDH Chemical LTD, Poole England) and standardized by adjusting to 0.5 McFarland Standard to produce final inocula of 1–5 × 10^8^ CFU/mL according to the Clinical and Laboratory Standards Institute (CLSI-M07A11) guidelines (Clinical and Laboratory Standards Institute [CLSI], 2018).

**TABLE 1:** Organisms used in the study.

Strain	Species	Relevant properties	Reference or Source
ATCC 25922	*Escherichia coli*	Sensitive to ampicillin: CLSI-recommended control organism for antimicrobial susceptibility testing	Selectrol, TCS Biosciences, United Kingdom.
LLH029E	*Escherichia coli*	Ampicillin-resistant	Fecal isolate (Molecular Biology Lab, University of Ibadan)
NCTC 6571	*Staphylococcus aureus*	Sensitive to penicillin, cloxacillin and ampicillin; CLSI-recommended control organism for antimicrobial susceptibility testing	Selectrol, TCS Biosciences, United Kingdom

### Test Compounds

Commercially procured powders of quinine sulfate (Sigma-Aldrich, United Kingdom), chloroquine phosphate (Sigma-Aldrich, United Kingdom), ampicillin sodium (Merck, United Kingdom), cloxacillin sodium monohydrate (Sigma-Aldrich, Germany), and nalidixic acid (Merck, United Kingdom) were used in the study. Test antibiotic solutions were prepared as outlined in the Clinical and Laboratory Standards Institute (CLSI) guideline. All test compounds were dissolved in water except nalidixic acid which was dissolved in 0.1 N sodium hydroxide (O’Neil, 2001). Stock solutions were prepared at 10 mg/mL for quinine and chloroquine and 1 mg/mL for ampicillin, cloxacillin, and nalidixic acid (Wiegand et al., 2008; Clinical and Laboratory Standards Institute [CLSI], 2018). Fresh stock solutions of ampicillin, cloxacillin and nalidixic acid was made for each experiment.

### Antimicrobial Susceptibility Testing

The antimicrobial activities of chloroquine, quinine and ampicillin were examined by determining the minimum inhibitory concentrations (MICs) and minimum bactericidal concentrations (MBCs) against the test organisms using the broth microdilution method as laid out in the CLSI M07-A11 guideline (Clinical and Laboratory Standards Institute [CLSI], 2018). The tests were performed in sterile, polystyrene 96-well round bottomed microtiter plates. Bacterial suspensions standardized by adjusting the turbidity with a spectrophotometer equivalent to a 0.5 McFarland standard (optical density of 0.08–0.13 at 625 nm, at 1-cm light path) were added to the wells of the microtiter plate containing 100 μL of twofold serial dilutions of the test antimicrobial to give final inoculum size of 5 × 10^5^ CFU/mL. Wells without the test organisms served as sterility control while inoculated wells without the drugs served as positive (growth) control. The plates were incubated at 37 °C for 24 h after which they were visually inspected and the OD_595_ was recorded using a microtiter plate reader. The MIC was defined as the lowest concentration of each drug resulting in complete inhibition of growth. The MBC was determined by sub-culturing the wells in the MIC microtiter plate into corresponding wells of a sterile microtiter plate containing 100 μL of Muller Hinton broth using a multi-point inoculator. The plate was then incubated at 37 °C for 24 h. The MBC was defined as the lowest concentration of each drug that inhibited bacterial growth compared to the untreated control culture, as shown by lack of turbidity in the wells. Not more than 2 microtiter plates were stacked in the incubator although CLSI allows up to 4 plates. Concentrations ranging from 7.8125 and 1,000 μg/mL were tested for chloroquine and quinine, and concentrations between 0.0122 and 50 μg/mL were tested for ampicillin and cloxacillin. Assays were performed in triplicates.

### Paper Strip Diffusion Test

This method is a qualitative approach to evaluating interactions between two compounds. To make ampicillin paper strips, ampicillin stock solution was prepared at 20 μg/mL in distilled water. Dilution of the stock solution was done to make a working solution of 2 μg/mL, from which 1 mL was added to individual 1 mm thick sterile Whatman filter paper strips (0.5 × 4 cm) to make ampicillin strips (2 μg). In a similar manner, chloroquine and quinine stock concentrations at 50 μg/mL were diluted to make 5 μg/mL working solutions, from which 1 mL was added to corresponding strips to make quinine strips (5 μg). When dry (after circa 2 h), the strips were placed adjoining but non-overlapping in a T-conformation on a MHA plate that had been surface-inoculated with a standardized suspension of the test organisms. Drugs were allowed to diffuse from the filter strips into the medium for 30 min at room temperature. The plates were then inverted and incubated at 35 ± 2 °C for 24 h. Plates with filter paper strips that had no drugs in them were used as growth controls. Commercially procured trimethoprim and sulfamethoxazole discs (Oxoid) arranged as strips in the T-conformation was used as a positive control for synergism (Bushby and Hitchings, 1968; Bernstein, 1982) and similarly placed ampicillin and trimethoprim disc combinations were used as a “no-interaction” control. The pattern of growth of test organisms was interpreted as follows: broadening of the zones of inhibition at adjoining ends depicts synergism, depression or narrowing of the zones indicates antagonism while no effect on the zones of inhibition indicate indifference (Laishram et al., 2017).

### Modified Disc Diffusion (MDD) Assay

In this method, disc diffusion tests are performed after incorporation of an agent in the agar medium to determine the nature of interactions between the agent in the medium and that in the disc (Amin et al., 2015; de Ruyck et al., 2016; Laishram et al., 2017). Doubling dilutions of the antimalarials (62.5–1,000 μg/mL) were made in molten Muller Hinton agar, which was then poured and set in plates aseptically. Bacteria inocula were standardized by adjusting the turbidity to 0.5 McFarland standard (OD_625_ at 0.08–0.13). The standardized inocula were spread on the agar surface according to the CLSI disc diffusion protocol (Clinical and Laboratory Standards Institute [CLSI], 2018) and ampicillin discs (10 μg) were applied. Mueller Hinton agar with no antimalarial drug was used as control. The plates were left at room temperature for about 1 h to allow diffusion of the antibiotic in the disc into the agar and then inverted and incubated at 35 ± 2 °C for 16–20 h. Diameters of zone of inhibition in millimeters were measured and plotted against log of chloroquine and quinine concentrations. In comparison with the inhibition zone of the ampicillin-only tests, an increase in bacterial zone diameter of ≥2 mm in the ampicillin-antimalarial containing disc is defined as synergy, an increase of <2 mm is considered weak synergy while a reduction in inhibition zone is defined as antagonism (Amin et al., 2015; Laishram et al., 2017). Assays were done in triplicate and repeated at least three independent times.

### Checkerboard Assay

The interactions between ampicillin and itself, chloroquine, quinine and nalidixic acid, and between cloxacillin and chloroquine and quinine against a selection of the test strains were studied using the checkerboard technique. To test, identical concentrations of ampicillin at 16 times its MIC, were added to the first well of each row and diluted twofold along the columns of a 96-well round- bottom microtiter plate (Greiner Bio-One, Germany). In a similar manner, concentrations of the antimalarial added to the top well in a column were diluted along the rows to give serial twofold dilutions. The two doubling dilutions were combined to yield a checkerboard with control wells on the lowest row and rightmost columns. Wells were challenged with the standardized inoculum, except for the sterility control wells, and the plates were incubated at 37 °C for 24 h. All tests were performed in triplicate. Optical density (OD_595_) readings were taken and the nature of interaction of the drug combinations were classified on the basis of the fractional inhibitory concentration index (FICI), i.e., the combination of ampicillin-antimalarial that produced the greatest change from ampicillin alone. The FICI is calculated for each strain and drug combination using the formula:

F⁢I⁢C⁢i⁢n⁢d⁢e⁢x⁢(F⁢I⁢C⁢I)=F⁢I⁢C⁢A+F⁢I⁢C⁢B

Where

*FICA* = *MIC of drug A in the combination/MIC of drug A alone*.

*FICB* = *MIC of drug B in the combination/MIC of drug B alone*.

Interactions were interpreted as synergistic if the FICI ≤ 0.5, additive if the FICI is > 0.5–1, indifferent if FICI > 1 < 2 and antagonistic if the FICI ≥ 2 (European Committee for Antimicrobial Susceptibility Testing (EUCAST) of the European Society of Clinical Microbiology and Infectious Diseases (ESCMID), 2000); Amin et al., 2015).

### Effect of Ampicillin-Antimalarial Combinations on Bacterial Cell Membrane Integrity

The leakage of potassium from the cells of test organisms was used to evaluate loss of membrane integrity caused by the ampicillin alone and when combined with quinine and chloroquine. The drug combination concentrations that led to a broadened inhibition zone in the disc diffusion assay was used. The test bacterial cells were grown in nutrient agar at 37 °C for 18 h and centrifuged at 3,700 rpm for 15 min. The cells were washed three times with 0.9 %w/v saline, re-suspended in 20 mL of the normal saline and challenged with individual drug solutions and drug combinations. The resulting solution was placed in an incubator-shaker at 37 °C for 24 h. After that, the cellular debris were separated by centrifugation at 3,500 rpm for 15 min and the supernatant was filtered through a 0.45 μm membrane filter. Aliquots of the supernatants were taken and stored in sample bottles which were frozen at −80 °C. The presence of potassium ions present in the samples were carried out using a flame photometer (PFP7, Jenway, Sweden) at wavelength of 766.480 nm. The instrument was calibrated using standard solutions containing 1, 5, 10, 15, and 20 μg/mL potassium chloride solutions. Chlorocresol was used as positive control while an inoculum suspension not treated with test compounds served as negative control. In all assays, the leakage of cellular components from bacteria into normal saline (blank) was subtracted from all samples.

### Statistical Analysis

Mean and standard deviations of replicates were summarized using Microsoft Excel while correlation analysis was used to find the relationship between drug concentration and inhibition zones at 5% level of significance. All variables represent mean values of three replicates.

## Results

### Quinine Possesses Antibacterial Activity and Both Chloroquine and Quinine Increase Antibacterial Activity of Ampicillin

Chloroquine did not demonstrate detectable antibacterial activity against either of the type cultures (MIC > 1,000 μg/mL) although decreasing turbidity was observed in the wells with increasing concentrations of chloroquine (Table 2). At chloroquine concentrations higher than 1,000 μg/mL, the drug’s solubility was poor. Quinine had MIC values of 1,000 μg/mL against *Escherichia* coli ATCC 25922 and *Staphylococcus aureus* NCTC 6571. We recorded MICs of 3.1 and 0.78 μg/mL for ampicillin against *E. coli* ATCC 25922 and *S. aureus* NCTC 6571, respectively. Cloxacillin had MICs of 100 and 0.19 μg/mL against *E. coli* ATCC 25922 and *S. aureus* NCTC 6571, respectively, and nalidixic acid had MIC values of 4 and 256 μg/mL against *E. coli* ATCC 25922 and *S. aureus* NCTC 6571, respectively. None of the penicillins tested showed activity against ampicillin-resistant isolate *E. coli* LLH029E (MIC > 100 μg/mL). Quinine inhibited this strain at 1,000 μg/mL.

**TABLE 2:** MIC of antibiotics and antimalarials against tested isolates using broth microdilution method (*n* = 3).

Drug tested	MIC and MBC of test drugs (μg/mL)						
	*E. coli* ATCC 25922		*S. aureus* NCTC 6571		*E. coli* LLH029E		*S. aureus* ATCC 29213
	MIC	MBC	MIC	MBC	MIC	MBC	MIC
Ampicillin	3.1	6.2	0.78	1.56	>100	ND	3.1
Cloxacillin	100	>100	0.19	0.390	>100	>100	NT
Nalidixic acid	4	8	256	512	NT	NT	64
Quinine	1,000	1,000	1,000	1,000	1,000	1,000	NT
Chloroquine	>1,000	>1,000	>1,000	>1,000	>1,000	>1,000	NT

**ND, means not determined; NT, not tested.**

As shown in Table 2, MBCs were twofold greater than respective MICs for all drugs except quinine, which was bactericidal at its MIC of 1,000 μg/mL.

**FIGURE 1:**
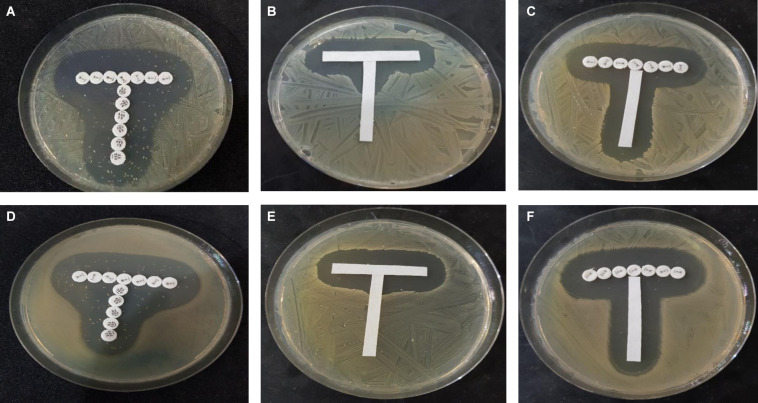
Paper strip diffusion test showing **(A,D)**, synergism between control antimicrobials trimethoprim (horizontally placed) and sulfamethoxazole (vertically placed); **(B,E)** potentiation of ampicillin strip (horizontal) by quinine (vertical); and **(C,F)** no interaction/slight inhibition between trimethoprim (horizontal) and ampicillin (vertical). **(A–C)** Show interaction against *S. aureus* NCTC 25922 while **(D–F)** shows interaction against *E. coli* ATCC 25922.

The paper strip diffusion method qualitatively illustrated interactions between the test compounds (Figure 1). Potentiation was observed with combinations of ampicillin and chloroquine and ampicillin and quinine: the zones of inhibition around ampicillin protruded vertically toward the strips containing chloroquine and quinine (chloroquine and quinine showed no activity at the concentrations tested). This protrusion was more marked with quinine against *S. aureus* NCTC 6571. Broadening of the inhibition zones of both trimethoprim and sulfamethoxazole, indicative of a synergistic effect, was observed with the positive control thus validating the experiment. In the negative control, the ampicillin and trimethoprim had well defined zones of inhibition indicating no interaction between the test compounds, this can be regarded as indifference.

**FIGURE 2:**
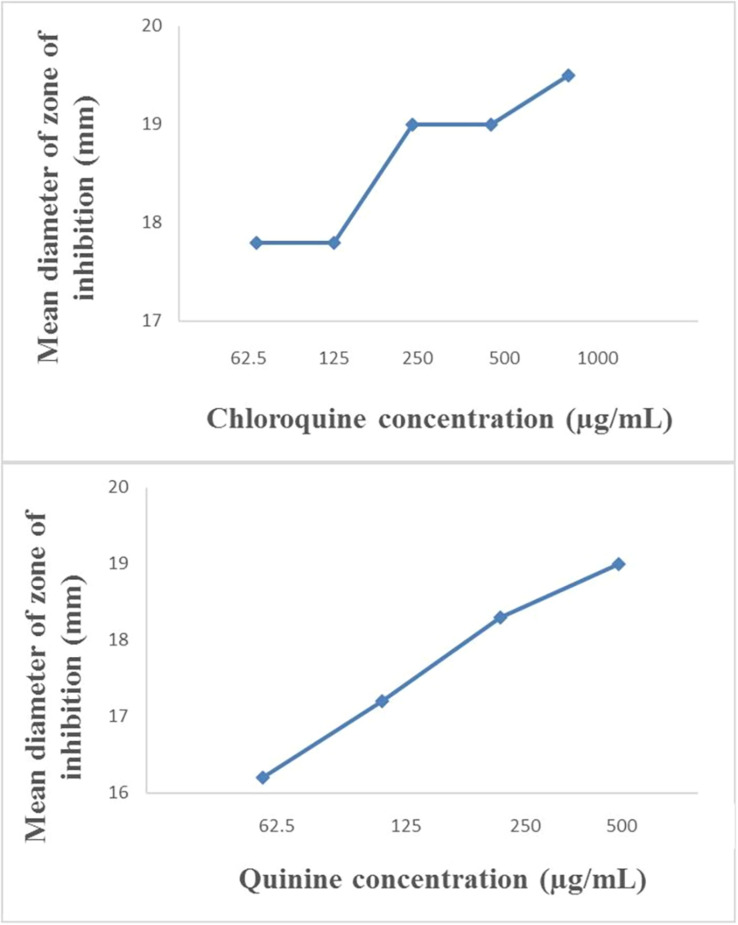
Plot of inhibition zone diameter against log of chloroquine and quinine concentration in combination with ampicillin (10 μg) using *E. coli* ATCC 25922 as test organism.

**FIGURE 3:**
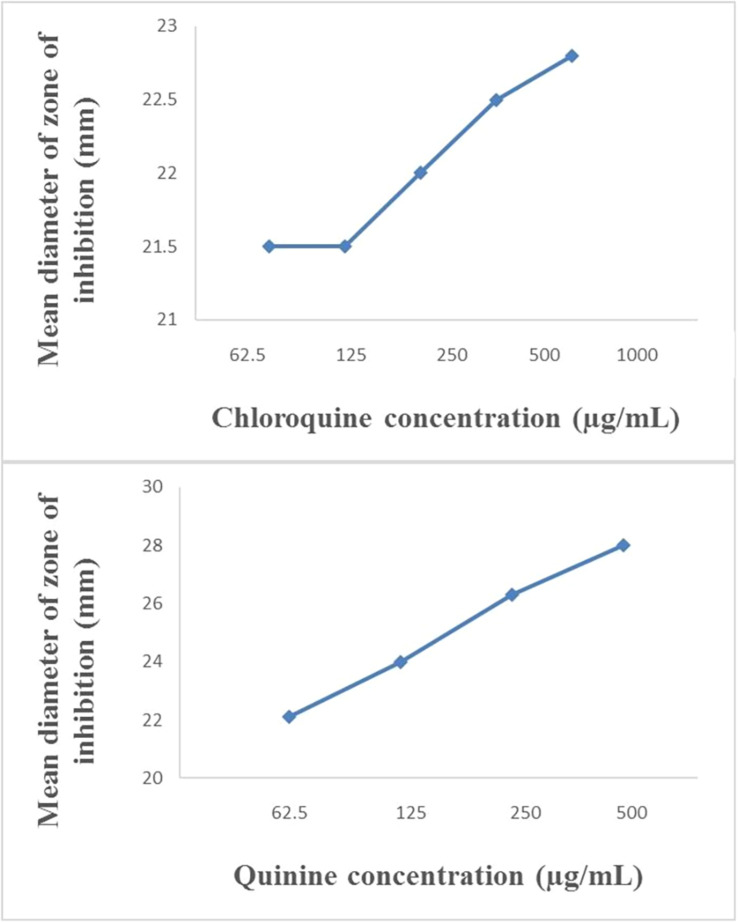
Plot of inhibition zone diameter against log of chloroquine and quinine concentration in combination with ampicillin (10 μg) using *S. aureus* NCTC 6571 as test organism.

The modified disc diffusion test showed a concentration-dependent increase in the zones of inhibition around ampicillin discs for both quinine and chloroquine (Figures 2, 3). The agar plates containing ampicillin disc only (no antimalarial embedded in the agar medium) had average zones of inhibition (mm) of 15.1 ± 0.1 and 19.2 ± 0.16 around the discs in agar inoculated with *E. coli* ATCC 25922 and *S. aureus* NCTC 6571, respectively. These zone diameter sizes indicate intermediate activity against *E. coli* ATCC 25922 and susceptibility for *S. aureus* NCTC 6571 according to the CLSI guideline (Clinical and Laboratory Standards Institute, 2014). The relationship between the square of the distance *d*^2^ (from the edge of the disc till the edge of the inhibition zone) and the log of the concentration of antimalarial in combination was linear with quinine for *E. coli* ATCC 25922 (*R*^2^ = 0.9946, *p* = 0.07) and for *S. aureus* NCTC 6571 (*R*^2^ = 0.9973, *p* = 0.04) and exponential with chloroquine for *E. coli* ATCC 25922 (*R*^2^ = 0.8814, *p* = 0.08) and for *S. aureus* NCTC 6571 (*R*^2 =^ 0.9444, *p* = 0.01). No zone diameter was reported for quinine at 1,000 μg/mL because growth was completely inhibited throughout the plate.

**FIGURE 4:**
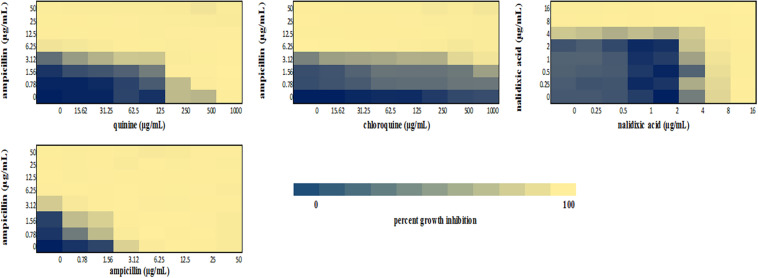
Checkerboard analysis of drug combinations tested against *E. coli* ATCC 25922. Data presented as a heatmap indicating percent growth inhibition based on OD_595_ values. Percent growth reduction values was calculated as 100% – [(OD of treated cells/OD of untreated cells) × 100%] (Ogundeji et al., 2017). Values represent mean values of three replicates.

**FIGURE 5:**
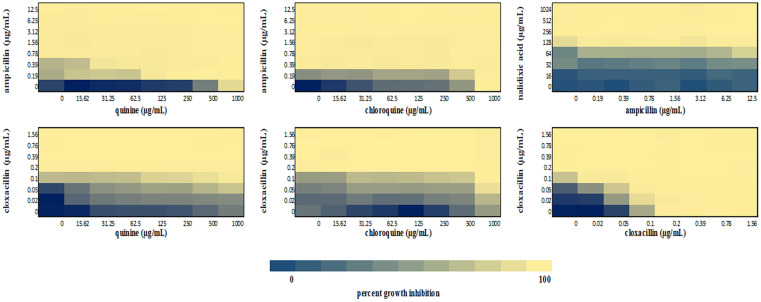
Checkerboard analysis of drug combinations tested against *S. aureus* NCTC 6571. Data presented as a heatmap indicating percent growth inhibition based on OD_595_ values. Percent growth reduction values was calculated as 100% – [(OD of treated cells/OD of untreated cells) × 100%] (Ogundeji et al., 2017). Values represent mean values of three replicates.

**FIGURE 6:**
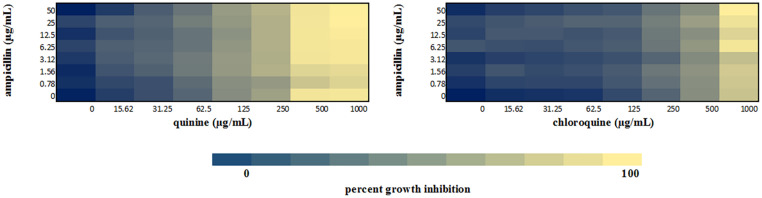
Checkerboard analysis of drug combinations tested against *E. coli* LLHO29E. Data presented as a heatmap indicating percent growth inhibition based on OD_595_ values. Percent growth reduction values was calculated as 100% –[(OD of treated cells/OD of untreated cells) × 100%] (Ogundeji et al., 2017). Values represent mean values of three replicates.

We performed a checkerboard experiment to more rigorously describe the ampicillin-quinolone interaction. Figures 4–6 display the interactions that were seen on a fine-scale, based on turbidity. They show that while the antimalarials did not fully inhibit growth at most of the test concentrations, they did have some inhibitory effects on their own and extended the inhibition of the penicillins. This was easily observed in the checkerboards of the sensitive strains (Figures 4, 5) but was also evident at the highest ampicillin dilution for the ampicillin resistant *E. coli* LLHO29E (Figure 6). FICIs were calculated based on absolute inhibition. The MICs in the checkerboard in non-combined wells were consistent with the values obtained from the independent broth dilution method reported in Table 2. The concentrations of ampicillin tested ranges from dilutions below and above the MIC but with quinine and chloroquine, the highest concentration tested was the MIC because above these concentrations, the drug did not completely dissolve in water.

The FICI values for ampicillin-quinine combinations were additive based on the interpretative criteria for both *E. coli* ATCC 25922 (FICI = 1.0) and *S. aureus* NCTC 6571 (FICI = 0.75). Additivity was observed with ampicillin-chloroquine combination against *E. coli* ATCC 25922 (FICI = 1.0) and indifference against *S. aureus* NCTC 6571, respectively (FICI = 1.25). *E. coli* LLH029E was fully resistant to both chloroquine and ampicillin and there was growth in all wells containing ampicillin-chloroquine combinations, although, turbidity hence optical density values decreased with increasing concentration of chloroquine (Figure 6). Quinine completely inhibited the growth of the *E. coli* LLH029E, an ampicillin resistant isolate, at its MIC but did not make the organism susceptible to ampicillin as growth was observed in wells containing combinations of ampicillin and quinine at concentrations of quinine lower than its MIC. There was, however, decreasing turbidity (and OD_595_ values) in wells as the concentration of quinine increased (Figure 6). Cloxacillin-quinine and cloxacillin-chloroquine combinations showed additive effects against *S. aureus* NCTC 6571 (FICI = 1.0 and 0.5, respectively). Ampicillin-nalidixic acid combinations showed indifference against *E. coli* ATCC 25922 and *S. aureus* NCTC 6571 (FICI = 1.5). Ampicillin-ampicillin combination was additive against *E. coli* ATCC 25922 (FICI = 0.75) and cloxacillin-cloxacillin combination was additive against *S. aureus* NCTC 6571 (FICI = 0.80) as expected with a drug in combination with equal concentrations of itself (Loewe, 1928).

### Observed Increase in Activity of Ampicillin by Antimalarials Is Likely Due to Unfettered Activity at Separate Targets

Since the checkerboard experiment revealed that the interaction between ampicillin and the different antimalarials was additive, the most likely explanation for it is independent activity at their separate targets. We, however, wanted to rule out, or otherwise, any possibility that the antimalarials were either increasing access of β-lactams to their target or directly affecting the activity of the cell wall active antibacterials. Significant potassium ion release was produced by chlorocresol (positive control) with both test organisms. As shown in Table 3, potassium ion leakage into the culture media from Gram-positive *S. aureus* NCTC 6571 mediated by ampicillin was not enhanced by the presence of any of the antimalarials. For Gram-negative *E. coli* ATCC 25922, neither ampicillin nor the antimalarials produced significant leakage alone or in combination. All in all, the data rule out a cell-integrity-centered mechanism for the antimalarial-ampicillin interaction.

**TABLE 3:** Cell permeability assay results (potassium leakage test) of drugs and drug combinations against quality control strains.

Test drug and drug combinations	Potassium release (mg/L)	
	*E. coli* ATCC 6571	*S. aureus* NCTC 6571
Ampicillin	1.5 ± 0.5	4
Quinine (1,000 μg/mL)	2	2
Chloroquine (1,000 μg/mL)	1.8 ± 0.17	2
Ampicillin + Quinine (125 μg/mL)	2.07 ± 0.81	4.33 ± 0.29
Ampicillin + Quinine (250 μg/mL)	1.67 ± 0.29	4.3
Ampicillin + Quinine (500 μg/mL)	1.33 ± 0.58	3.5 ± 0.5
Ampicillin + Quinine (1,000 μg/mL)	1.17 ± 0.29	3.83 ± 0.29
Ampicillin + Chloroquine (125 μg/mL)	1	4
Ampicillin + Chloroquine (250 μg/mL)	1	3.33 ± 0.58
Ampicillin + Chloroquine (500 μg/mL)	1	3.33 ± 0.58
Ampicillin + Chloroquine (1,000 μg/mL)	1	3.5 ± 0.5
Chlorocresol	5	6.67 ± 0.29

**Data are mean ± SD, or mean where SD = 0.**

## Discussion

Malaria can cause immune suppression, leaving malaria-burdened patients prone to bacterial infections (O’Dempsey, 2000; Adesanmi et al., 2011; Falade et al., 2016). Clinical co-administration is common in malaria endemic areas, prompting some studies of interactions, which have yielded reports of significant antagonistic drug-drug interactions between penicillin antibiotics and antimalarials when taken concurrently in healthy populations (Ali, 1985; Babalola et al., 2002; Babalola et al., 2003; Babalola et al., 2009; Falade et al., 2016).

Falade et al. (2016) reported an increase in the MIC and MBC of ampicillin and cloxacillin in the presence of quinine against *Staphylococcus aureus* (Falade et al., 2016). This study, however, did not use media recommended for MIC testing, had very wide confidence intervals, did not test for the antibacterial activity of quinine and did not establish a concentration-dependent interaction between the two agents. Thus, while suggested mechanisms of pharmacokinetic interactions in the reported *in-vivo* studies are plausible, no published report has adequately described the nature of any interactions or rigorously confirmed the antagonism *in vitro.* In contrast to Falade et al. (2016) one earlier *in vitro* study found no interaction or slight potentiation of ampicillin by quinine (Abreu et al., 2014). For these reasons, a more rigorous exposition of the antibacterial interactions was performed in this study.

The MIC values of ampicillin and nalidixic acid against *E. coli* ATCC 25922 (3.1 and 4 μg/mL, respectively) were consistent with those reported in the literature (Andrews, 2001; Kingdom and Dickinson, 2002; Clinical and Laboratory Standards Institute [CLSI], 2018), as was the MIC of cloxacillin which was below 2 μg/mL (0.19 μg/mL) (Matynia et al., 2005; Al-Harbi et al., 2017; Buldain et al., 2018). The MIC value of 256 μg/mL obtained with nalidixic acid against *S. aureus* NCTC 6571 is consistent with that reported by Andrews (2001) (>128 μg/mL) while the MIC of ampicillin against *S. aureus* NCTC 6571 (0.78 μg/mL) is within the range of 0.06–2 μg/mL reported in literature (European Committee for Antimicrobial Susceptibility Testing [EUCAST], 2000; Andrews, 2001; Adeleke and Olaitan, 2010; Jeyaseeli et al., 2012; Fratini et al., 2017; Rishi et al., 2018).

In this study, quinine showed detectable antibacterial activity against the three organisms used in the study with an MIC of 1,000 μg/mL against *E. coli* ATCC 25922, *S*. *aureus* NCTC 6571, and *E. coli* LLH029E. The MIC falls within the reported range of values (Kharal et al., 2009; Abreu et al., 2014). Our data show that quinine is bactericidal at 1,000 μg/mL and while this concentration is unlikely to be achieved physiologically during treatment, the additivity uncovered in this work indicates that lower concentrations may produce therapeutic effects in combination with antibacterials. The same is possibly true for chloroquine for which we could not record an MIC in this study because higher concentration could not be solubilized. We did see decreasing turbidity in wells with increasing concentrations of chloroquine (Figures 4–6), indicating some antibacterial properties. Studies have also reported antibacterial activity of chloroquine: an early study reported a pH dependent inhibition of exponential growth of *E. coli* cultures by chloroquine (Wiseman, 1972; Middleton and Wiseman, 1974). Another study reported inhibition zones with chloroquine concentrations as low as 30 μg (Jagadeesh et al., 2014) and more recently, MIC values ranging between 625 and 1,200 μg/mL against susceptible *E. coli* isolates and between 5,000 and 80,000 μg/mL against ciprofloxacin resistant isolates have been reported (Davidson et al., 2008). This is unsurprising since quinolone class of antibacterial drugs were first discovered as by-products from the synthesis of chloroquine (Naeem et al., 2016).

Broadening of inhibition zones around the contact point of the two strips embedded with different agents is often seen in synergism, where both agents are active, or potentiation when one agent is active in the paper strip diffusion test we employed (Lorian and Fodor, 1974; Laishram et al., 2017). Our paper-strip test did indicate that quinine and chloroquine potentiated the activity of ampicillin but as the test is qualitative and has not been widely evaluated, we performed other experiments to study the interactions.

The disk diffusion test modified for testing interactions between drug combinations was first described by Climo et al. (1999) where they determined its validity for uncovering synergy of combinations of vancomycin and beta-lactam antibiotics against staphylococci with reduced susceptibility to vancomycin (Climo et al., 1999). It is now commonly used to study drug interactions (Kiraz et al., 2010; Abreu et al., 2014; Amin et al., 2015; Sy et al., 2016) and the presence of interactions is subject to the method of interpretation. Some interpretations include a weak synergy, defined as a <2 mm increase in zone (Laishram et al., 2017), this definition might fit more appropriately to combinations of ampicillin and quinine on ATCC 25922 where a +1 mm increase in zone diameter was observed if juxtaposed with results of the paper strip assay where potentiation was observed. An increase in inhibition zone by ≥ 2 mm indicates synergism or at the least additivity between ampicillin and quinine on *S. aureus* and at the MIC of quinine, total synergy occurred in both isolates, demonstrated by complete inhibition of growth throughout the entire agar surface. A resulting straight line when the square of zone diameter is plotted against increasing log concentrations of quinine ratifies the interpretation (Figures 2, 3). Combinations of ampicillin and chloroquine against the two isolates may be better than indifferent, since the relationship between the square of inhibition zone sizes and increasing log concentrations was exponential (Figures 2, 3), similar to some previous reports (Jain, 2003; Abreu et al., 2014; Jagadeesh et al., 2014).

The gold standard checkerboard method was lastly used to characterize the type of interaction between ampicillin-quinine, and ampicillin-chloroquine combinations on *E. coli* ATCC 25922, *S. aureus* NCTC 6571, and *E. coli* LLH029E. Since ampicillin is often combined with cloxacillin in dosage forms to reduce resistance against penicillinase-producing Gram-positive bacteria (Martindale, 2011), the interaction between cloxacillin and chloroquine, as well as cloxacillin and quinine was studied in *S. aureus* NCTC 6571 (Moody, 2002; Martindale, 2011; Farrington, 2012). Ampicillin concentration ranges between 16 and 1/4 times the MIC were tested but the highest quinine concentration tested was at the MIC because over this concentration, supersaturation of the drug in its solvent caused the drug to crystallize out obscuring reading of the results. Since chloroquine did not show antibacterial activity indicated by complete inhibition of growth the same concentration range was used for both chloroquine and quinine.

Our checkerboard experiment allowed for simultaneous determination of MIC and FIC on the same microtiter plate and therefore the same dilutions of drugs and test organisms, allowing variations to affect the determination the same way, even among replicates (Fratini et al., 2017). This is often not accounted for in methods that offer more strict interpretive guidelines (White et al., 1996; Odds, 2003). The checkerboard assay results revealed additivity between ampicillin and the quinoline antimalarials tested. This corroborates the results of the MDD assay and paper strip diffusion tests as well as the earlier report of Abreu et al. (2014).

The most logical explanation for additive activity is that each agent exerts its activity without interfering with the mechanism of the other. Because paper-strip and MDD activities suggested that some potentiation or synergism was possible, we sought to determine whether the antimalarials affected membrane integrity or operated in any way at the penicillin target.

Damage to cell membranes, which is often secondary to cell wall disruption, is characterized by discernable leakage of cytoplasmic constituents, especially low molecular weight constituents such as potassium ions (Abbanat et al., 1998; Amarnath et al., 2003; Epstein, 2003; Johnston et al., 2003; Amin et al., 2015; El-Batanony, 2017). Quinine and chloroquine alone, and in combination with ampicillin did not result in appreciable amounts of potassium leakage even at the MIC of quinine (Table 3). This suggests that induction of cell leakage is not responsible for the observed additive effects of these antimalarials with ampicillin.

Damage to cell membranes, which is often secondary to cell wall disruption, is characterized by discernable leakage of cytoplasmic constituents, especially low molecular weight constituents such as potassium ions (Abbanat et al., 1998; Amarnath et al., 2003; Johnston et al., 2003; Amin et al., 2015; El-Batanony, 2017). Quinine and chloroquine alone, and in combination with ampicillin did not result in appreciable amounts of potassium leakage even at the MIC of quinine (Table 3). This suggests that induction of cell leakage is not responsible for the observed additive effects of these antimalarials with ampicillin.

## Conclusion

In conclusion, our investigations reveal that chloroquine and quinine have some antibacterial activity and yield at least additive effects at high concentrations when combined with ampicillin and most likely other penicillins (interaction with cloxacillin also yielded additivity) *in vitro*. The data clearly refute antagonism between the two drug classes. The use of chloroquine and quinine in the treatment of malaria may therefore offer an additional advantage of preventing or curing bacterial infections, even against resistant isolates provided that they are not counteracted by interactions at the biopharmaceutic or pharmacokinetic level. Increased antibacterial activity may especially be achievable in non-oral formulations where no antagonistic interactions between antimalarials and penicillins have been reported. For orally administered drugs, it is possible that the additive activity could, at the very least, counterbalance the earlier reported negative effects of quinolone antimalarials on ampicillin pharmacokinetics (Falade et al., 2016). Additivity likely arises from mutual non-interference of antibacterial activity. We have been able to rule out cell leakage as the means by which quinine and chloroquine exert their additive effects with ampicillin. These antimalarials therefore likely exert their additive effects without interacting with the mechanism of antibacterial action of ampicillin. Further *in vivo* investigations are recommended to determine whether this is a subtle synergistic effect, and if so, any mechanism by which this occurs. Lack of interaction between chloroquine/quinine and nalidixic acid combinations suggest that inhibition of DNA gyrase may not be the mechanism of additive interactions and other mechanisms should be investigated. It would also be worth testing other antibacterial-antimalarial combinations, particularly those that are in greater use in today’s clinics.

## Data Availability Statement

The raw data supporting the conclusions of this article will be made available by the authors, without undue reservation.

## Author Contributions

OAO performed literature review, performed, analyzed and interpreted experiments, provided resources, and drafted the manuscript. CB conceived the project, contributed to literature review, co-supervised OAO, interpreted data, and performed critical review. OOO designed and supervised *in silico* studies, provided resources, performed, analyzed and interpreted experiments, and wrote a section of the manuscript. OK co-supervised OAO and performed critical review. DK contributed to the experimental design, analysis, and supervision. AO performed and analyzed and interpreted experiments. IO designed, analyzed and interpreted experiments, performed literature searches, co-supervised OAO and DK, provided resources, performed critical reviews and contributed significantly to writing. All authors contributed to writing and approved the final draft.

## Conflict of Interest

The authors declare that the research was conducted in the absence of any commercial or financial relationships that could be construed as a potential conflict of interest.
